# Development of Specific Monoclonal Antibodies against Porcine RIG-I-like Receptors Revealed the Species Specificity

**DOI:** 10.3390/ijms24044118

**Published:** 2023-02-18

**Authors:** Qi Shao, Shuangjie Li, Qi Cao, Haotian Gu, Jiajia Zhang, Youwen Zhang, Kaili Zhang, Wanglong Zheng, Nanhua Chen, Shaobin Shang, Jianzhong Zhu

**Affiliations:** 1 College Veterinary Medicine, Yangzhou University, Yangzhou 225009, China; 2 Joint International Research Laboratory of Agriculture and Agri-Product Safety, Yangzhou University, Yangzhou 225009, China; 3 Comparative Medicine Research Institute, Yangzhou University, Yangzhou 225009, China; 4 Jiangsu Co-Innovation Center for Prevention and Control of Important Animal Infectious Diseases and Zoonoses, Yangzhou University, Yangzhou 225009, China

**Keywords:** RLRs, porcine, human, monoclonal antibody, species specificity

## Abstract

The RIG-I-like receptors (RLRs) play critical roles in sensing and combating viral infections, particularly RNA virus infections. However, there is a dearth of research on livestock RLRs due to a lack of specific antibodies. In this study, we purified porcine RLR proteins and developed monoclonal antibodies (mAbs) against porcine RLR members RIG-I, MDA5 and LGP2, for which one, one and two hybridomas were obtained, respectively. The porcine RIG-I and MDA5 mAbs each targeted the regions beyond the N-terminal CARDs domains, whereas the two LGP2 mAbs were both directed to the N-terminal helicase ATP binding domain in the Western blotting. In addition, all of the porcine RLR mAbs recognized the corresponding cytoplasmic RLR proteins in the immunofluorescence and immunochemistry assays. Importantly, both RIG-I and MDA5 mAbs are porcine specific, without demonstrating any cross-reactions with the human counterparts. As for the two LGP2 mAbs, one is porcine specific, whereas another one reacts with both porcine and human LGP2. Thus, our study not only provides useful tools for porcine RLR antiviral signaling research, but also reveals the porcine species specificity, giving significant insights into porcine innate immunity and immune biology.

## 1. Introduction

The innate immune system works as the first line of host immune defense, mainly relying on the pattern recognition receptors (PRRs) to sense various danger signals from pathogens that are called pathogen-associated molecular patterns (PAMPs) [1]. PRRs include, but not limited to, Toll-like receptors (TLRs), RIG-I-like receptors (RLRs), NOD-like receptors (NLRs), C-type lectin-like receptors (CLRs) and cytosolic DNA receptors (CDRs). Upon activation by PAMPs, the PRRs trigger intracellular signaling, and in turn, induce anti-viral interferons (IFNs), proinflammatory cytokines and chemokines, which together, directly combat pathogens and influence subsequent adaptive immunity [1]. 

Among the various PRR families, RLRs are the family of DExD/H-box helicases responsible for the detection of cytosolic viral RNA, and they encompass RIG-I, MDA5 and LGP2 [2]. Both RIG-I and MDA5 have a similar structural architecture: an N-terminal signal-active two-tandem caspase recruitment domain (CARDs), a middle DExD/H-box helicase domain and a C-terminal repressor domain (CTD) or a regulatory domain (RD) [3,4]. The optimal RNA agonist recognized by RIG-I is the 5’-ppp dsRNA (double stranded RNA) without the 5′-terminal nucleotide 2’-O-methylation, whereas MDA5 has no RNA end specificity and prefers internally binding to the long dsRNA [5,6]. Upon engagement by dsRNA, RIG-I and MDA5 recruit and activate downstream common adaptor MAVS. Then, the aggregated MAVS relays the signaling to TRAF3/TBK1/IKKε and TRAF6/IKKs, which activate transcription factors IRF3 and NF-κB, and drive IFN and proinflammatory cytokine expression, respectively. Thus, the antiviral status is achieved [7].

LGP2, as the third member of the RLRs, harbors similar helicase and CTD domains such as RIG-I and MDA5, but it lacks the N-terminal signaling CARDs. Although LGP2 itself is inactive in signaling, it has a stronger ability to bind with dsRNA and enables to regulate RIG-I and MDA5 activity [8]. In spite of the controversial role of LGP2 in the regulation of RIG-I and MDA5 signaling [5,9,10,11], our recent work showed that porcine LGP2 is able to positively regulate RIG-I and MDA5 signaling by promoting dsRNA binding and exerts an antiviral role against several porcine viruses [12]. 

Unlike TLRs, RLRs are expressed almost in all nucleated cells and play universal roles in RNA virus recognition and immune responses [3,13]. Considering the key role of RLRs in antiviral innate immunity, RLRs and the triggered immune signals have been extensively studied. However, due to the lack of antibody tools, there are a dearth of systemic investigations of animal RLRs. Porcine (*Sus scrofa*, pig) is an important livestock species, as well as a promising animal model to study several human diseases including infectious diseases [14,15]. Accumulating evidence has showed the essential roles of RLRs in the antiviral function against different porcine viruses [16,17,18,19,20]. In this study, we sought to develop the monoclonal antibodies (mAbs) against porcine RLRs, RIG-I, MDA5 and LGP2, and the obtained mAbs were successfully subjected to various immune assays. Importantly, the availability of porcine RLR-specific mAbs demonstrates the species specificity between the pig and human RLRs. 

## 2. Results

### 2.1. Porcine RLR Protein Purification and Development of RLR-Specific Hybridoma

**Figure 1:**
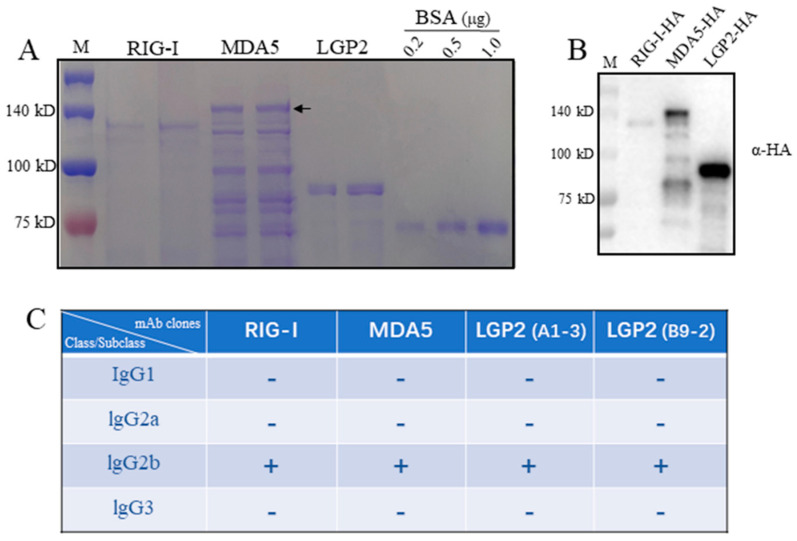
The porcine RLR purified proteins and the isotypes of four monoclonal antibodies against RLRs. (**A**) The purified proteins of porcine RLRs, RIG-I, MDA5 and LGP2, together with control BSA protein were subjected to SDS-PAGE and stained with Coomassie brilliant blue. The arrow head indicates the full-length MDA5. (**B**) The pRIG-I, pMDA5 and pLGP2 proteins were subjected to SDS-PAGE, transferred to PVDF membrane and detected by immunoblotting with anti-HA mAb. The protein markers are labeled on the left. (**C**) With the mouse monoclonal antibody Ig class/subclass/subtype identification kit, 4 strains of porcine RLR monoclonal antibodies derived from ascites all belong to IgG2b.

Porcine RIG-I, MDA5 and LGP2 expressed in bacteria were purified, in which RIG-I and MDA5 were from the supernatant of bacteria lysates as soluble proteins, whereas LGP2 was from a precipitate of bacteria lysate as an inclusion body. In SDS-PAGE, the RIG-I and LGP2 proteins exhibit as the major bands, which are 120 kD and 80 kD, respectively (Figure 1A). As for the MDA5 protein, multiple bands appear, with the largest one (> 140 kD) representing the full-length MDA5 (Figure 1A). All three RLR proteins carry C-terminal HA tags, and accordingly, the purified RLR proteins were recognized by anti-HA in Western blotting, as expected (Figure 1B).

**Figure 2:**
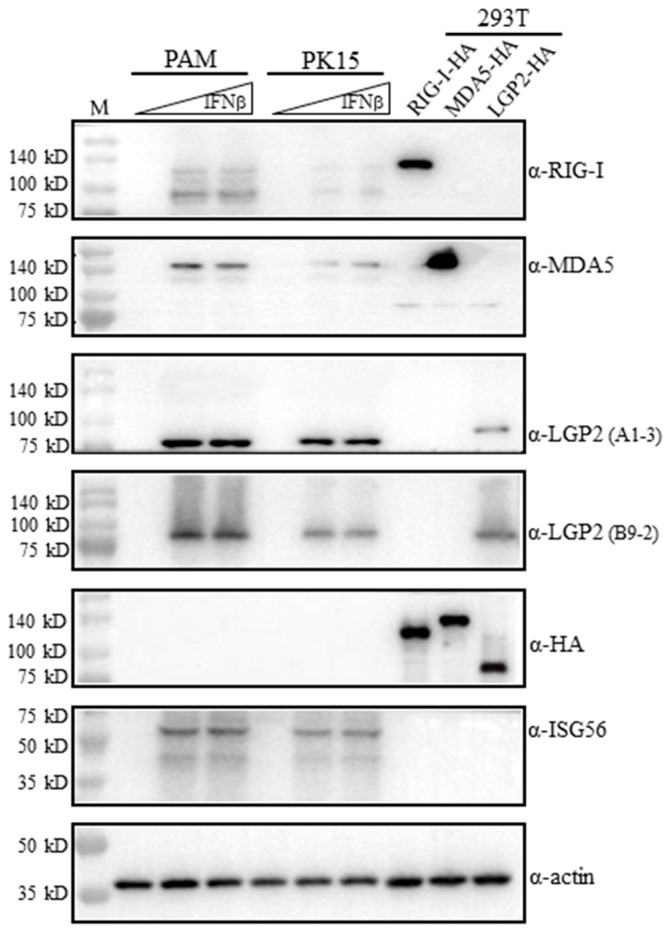
The reactivity of RLR mAbs with exogenous and endogenous porcine RLR proteins in Western blotting. 293T cells grown in 24-well plates (3 × 10^5^ cells/well) were transfected with HA tagged pLGP2, pRIG-I, pMDA5 or pcDNA (0.5 μg each) using Lipofectamine 2000 for 24 h. PAM cells and PK15 cells were plated on 24-well plates (2 × 10^5^ cells/well) and stimulated with human IFN-β (500 IU/mL, 1000 IU/mL) for 8–12 h. The above cell lysates were analyzed by Western blotting using the pRIG-I, pMDA5 and pLGP2 ascite mAbs, ISG56 pAb, HA mAb and Actin mAb (1:1000 dilution each). The protein markers are labeled on the left.

The spleens of immunized mice were fused with mouse myeloma Sp2/0, and the hybridomas were screened by indirect ELISA. The hybridomas with continuous, positive ELISA of more than three times were further validated by Western blotting (Figure 2). Finally, one, one and two monoclonal hybridomas were obtained for porcine RIG-I, MDA5 and LGP2, respectively. Based on the immunoglobulin class and subclass typing assay, all of the monoclonal antibodies (mAbs) from these four hybridomas were IgG2b subclasses (Figure 1C). 

### 2.2. Characterization of mAb Reactivity with Porcine RLRs by Western Blotting

On the one hand, the mAbs were examined for reactivity with porcine RLRs in the transfected 293T cells. The transfected porcine RIG-I, MDA5 and LGP2 all carry C-terminal HA tags, and their expressions in transfected cells were verified by anti-HA mAb (5th blot, Figure 2). The ectopic porcine RIG-I, MDA5 and LGP2 could be detected by anti-RIG-I mAb, anti-MDA5 mAb and anti-LGP2 mAbs (A1-3 and B9-2 clones), respectively (1st–4th blots, Figure 2). However, it should be noted that each RLR mAbs only reacted with its own members, and they did not cross-react with other RLR members (1st–4th blots, Figure 2). 

On the other hand, the mAbs were tested for reactivity with endogenous porcine RLRs in porcine alveolar macrophages (3D4/21, PAM) and PK15 cells. The RLRs belong to IFN-stimulated genes (ISGs) and normally are lowly expressed in quiescent cells. Both PAM and PK15 were stimulated with IFNβ to induce significant ISG expressions for detection, and downstream ISG56 proteins were indeed produced upon stimulation, as expected (6th blot, Figure 2). The induced porcine RIG-I, MDA5 and LGP2 were detected by anti-RIG-I mAb, anti-MDA5 mAb and anti-LGP3 mAbs (A1-3 and B9-2 clones), respectively (1st–4th blots, Figure 2). The endogenous RIG-I and LGP2 detected by mAbs are a little bit smaller than the ectopic proteins are, but endogenous MDA5 is identical in size to the ectopic protein, which is likely due to its higher molecular weight (>140 kD) (1st–4th blots, Figure 2). Further, there exist additional small proteins during both RIG-I mAb and MDA5 mAb detection (1st–2nd blots, Figure 2).

### 2.3. Immunofluorescence Using mAbs Revealed the Distinct Cytoplasmic Localizations of Porcine RLRs

**Figure 3:**
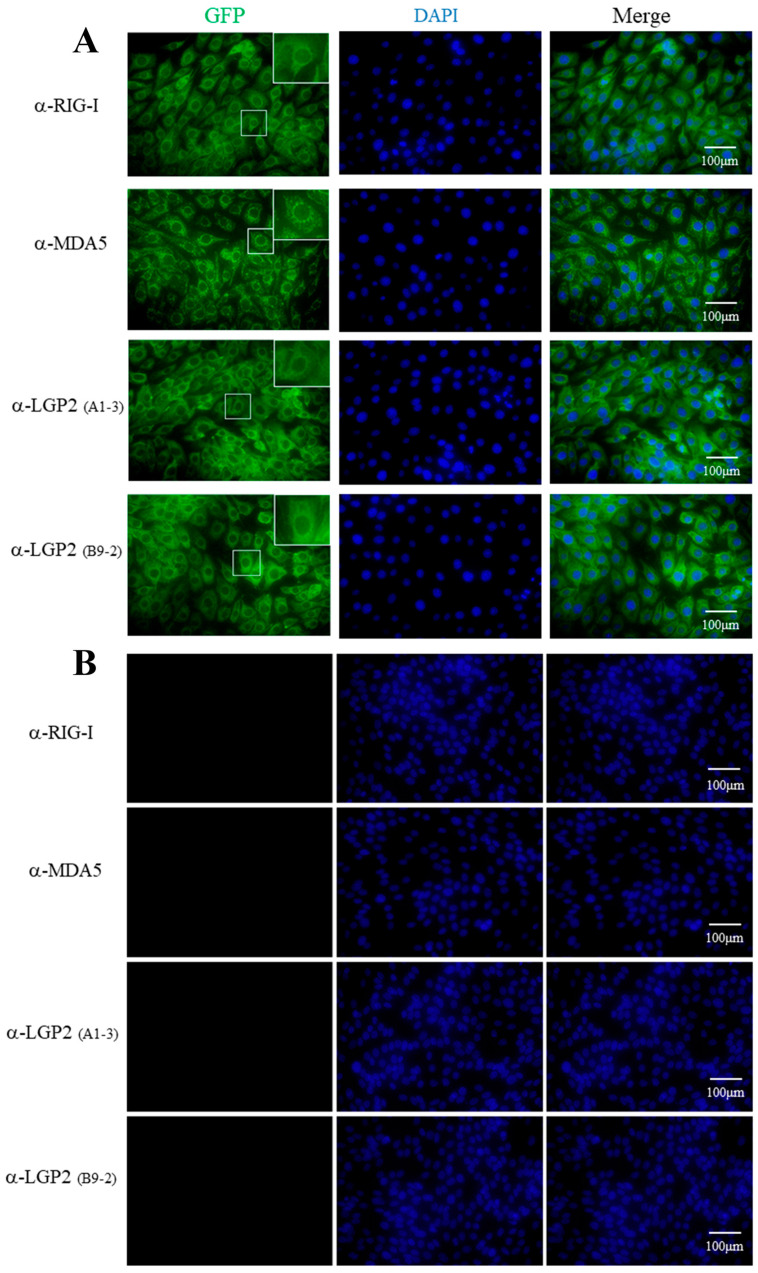
The cellular localizations of porcine RLRs identified by RLR mAbs in immunofluorescence assay (IFA). The PAM cells in 15 mm glass bottom cell culture dishes were grown to 70–80% and stimulated with 500 IU/mL human IFN-β for 8-12 h (**A**) or without stimulation (**B**). The cells were fixed, permeabilized and incubated with pRIG-I, pMDA5 and pLGP2 ascite mAbs (1:200 each) at 4°C overnight. Then, the cells were stained with secondary antibody Alex Fluor^TM^ 488 Donkey anti-Mouse Antibody (1:800) and counterstained with DAPI. The cellular localizations of pRIG-I, pMDA5 and pLGP2 in the PAM cells were examined under a fluorescence microscope. The boxed areas are magnified and shown on the upper right corners of each panels in (**A**). There was no reaction of non-stimulated PAM cells with RLR mAbs (**B**).

Four mAbs were utilized to detect cellular RLRs in PAM stimulated with IFNβ by indirect immunofluorescence (IFA). As shown in Figure 3, both the RIG-I mAb and LGP2 mAbs (A1-3 and B9-2 clones, respectively) mainly recognized the cytoplasmic proteins with a similar pattern, i.e., the recognized fluorescent proteins were distributed continuously in cytosol, especially in the perinuclear regions. Although MDA5 mAb also recognized the cytoplasmic protein, the recognized fluorescent proteins were distributed discontinuously, forming numerous puncta in cytosol, including in the perinuclear regions. The underlying reason for the different cytosol pattern of MDA5 localization from those of RIG-I and LGP2 is unknown, and it needs to be further confirmed. 

### 2.4. Determination of the Recognition Regions on Porcine RLRs and Cross-Reactivity with Human RLRs by mAbs

**Figure 4:**
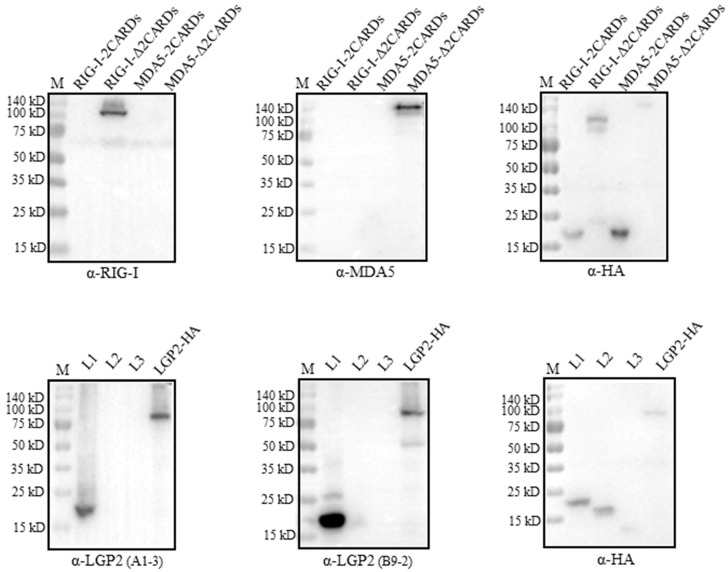
The identification of regions on three porcine RLR proteins recognized by four RLR mAbs. We transfected 293T cells (2 × 10^5^ cells/well) in 24-well cell culture plates with HA-tagged expression plasmids for pRIG-I-CARDs, pRIG-I-ΔCARDs, pMDA5-CARDs, pMDA5-ΔCARDs, pLGP2-L1, pLGP2-L2 and pLGP2-L3 (0.5 μg each) for 48 h, and the harvested cell lysates were subjected to Western blotting with HA mAb and the indicated porcine RLR ascite mAbs. The protein markers are labeled on the left.

The recognition regions by RLR mAbs were analyzed by Western blotting using the truncated porcine RLR proteins. The porcine RIG-I and MDA5 truncated proteins, including the N-terminal CARDs andΔCARDs, were confirmed to be expressed by the detection of a tagged HA (upper right panel, Figure 4). Neither RIG-I mAb nor MDA5 mAb recognized its own N-terminal CARDs region, instead, both mAbs recognized the regions beyond the CARDs (ΔCARDs) (upper left and middle panels, Figure 4). As for the two LGP2 mAbs, both mAbs recognized the N-terminal helicase ATP binding domain (L1, 1-200 aa), while the three porcine LGP2 fragments were all expressed by the detection of a tagged HA (lower panels, Figure 4).

**Figure 5:**
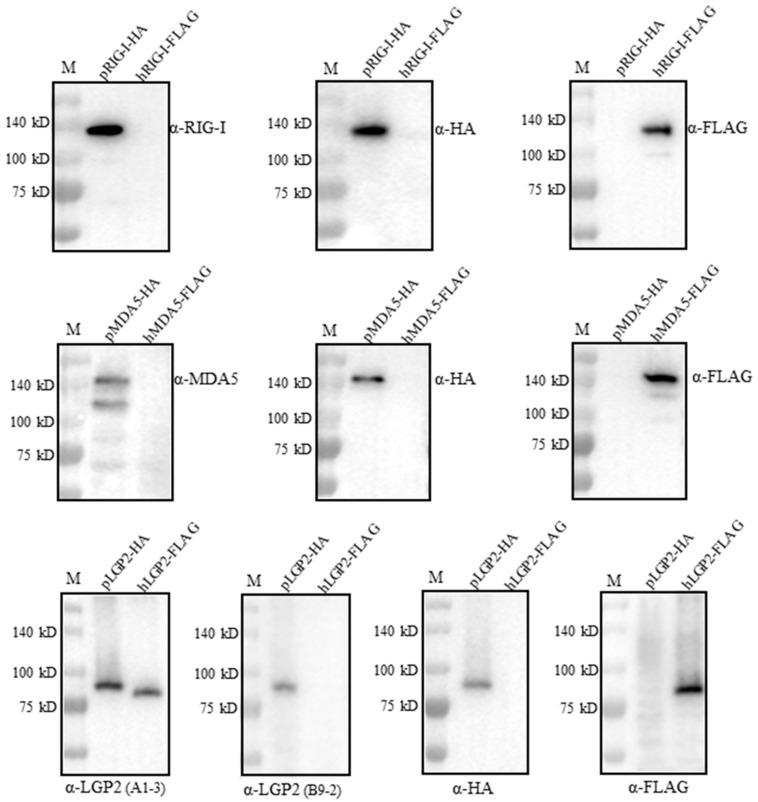
Examination of the cross-reactivity with human RLR proteins by the four porcine RLR mAbs. We transfected 293T cells (2 × 10^5^ cells/well) in 24-well cell culture plates with expression plasmids including pcDNA-pRIG-I-2HA, pcDNA-pMDA5-2HA, pcDNA-pLGP2-2HA and pcDNA-hRIG-I-3FLAG, pcDNA-hMDA5-3FLAG and pcDNA-hLGP2-3FLAG (1 μg each) for 24 h. The cell lysates were analyzed by Western blotting. The porcine RLR protein expressions were confirmed by HA mAb, whereas human RLR protein expressions were confirmed by FLAG mAb. The protein markers are labeled on the left.

We wondered if the four RLR mAbs could cross-react with the human RLR counterparts. For this purpose, FLAG-tagged human RIG-I, MDA5 and LGP2 were expressed in the transfected 293T cells, HA-tagged porcine RIG-I, MDA5 and LGP2 were expressed in the transfected 293T cells, and all of the RLR protein expressions were confirmed by the detection of the tag expressions (Figure 5). Interestingly, both RIG-I mAb and MDA5 mAb only reacted with porcine RLRs, but not the human RLR proteins (upper and middle panels, Figure 5). Similarly, LGP2 mAb (clone B9-2) only reacted with porcine LGP2, but not human LGP2; however, LGP2 mAb (clone A1-3) reacted with both porcine LGP2 and human LGP2 (lower panel, Figure 5). Thus, one set of porcine RLR specific mAbs was obtained in our study.

### 2.5. Detection of RLR Expressions in Different Tissues from PRRSV-Infected Pigs Using mAbs

**Figure 6:**
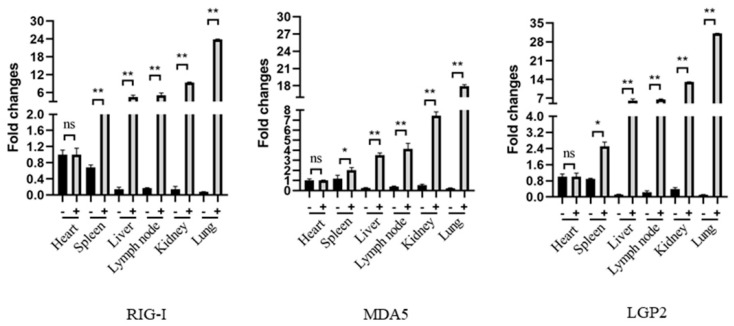
The expressions and distributions of RLR gene transcripts in various pig tissues in RT-qPCR. The heart, liver, spleen, lung, kidney and lymph node tissues were collected from pigs infected with HP-PRRSV (+) and non-infected control pigs (-), and the total RNA in each tissue was extracted to synthesize cDNA by reverse transcription. The RLRs, RIG-I, MDA5 and LGP2, gene expressions in these tissues were analyzed by qPCR. The three RLR genes exhibited similar patterns. * *p* < 0.05, ** *p* < 0.01 vs. normal tissues.

Before the Immunohistochemistry (IHC) analysis, the RLR mRNA expressions in the pig tissues were first analyzed by RT-qPCR, and the results showed that all of the tissues except those of the heart have a significant upregulation of RIG-I, MDA5 and LGP2 mRNA expressions upon PRRSV infection relative to those of the normal tissues. In the PRRSV tissues, the lung and kidney tissues have highest RLR mRNA levels, followed by the lymph node, liver, spleen and heart (Figure 6). 

**Figure 7:**
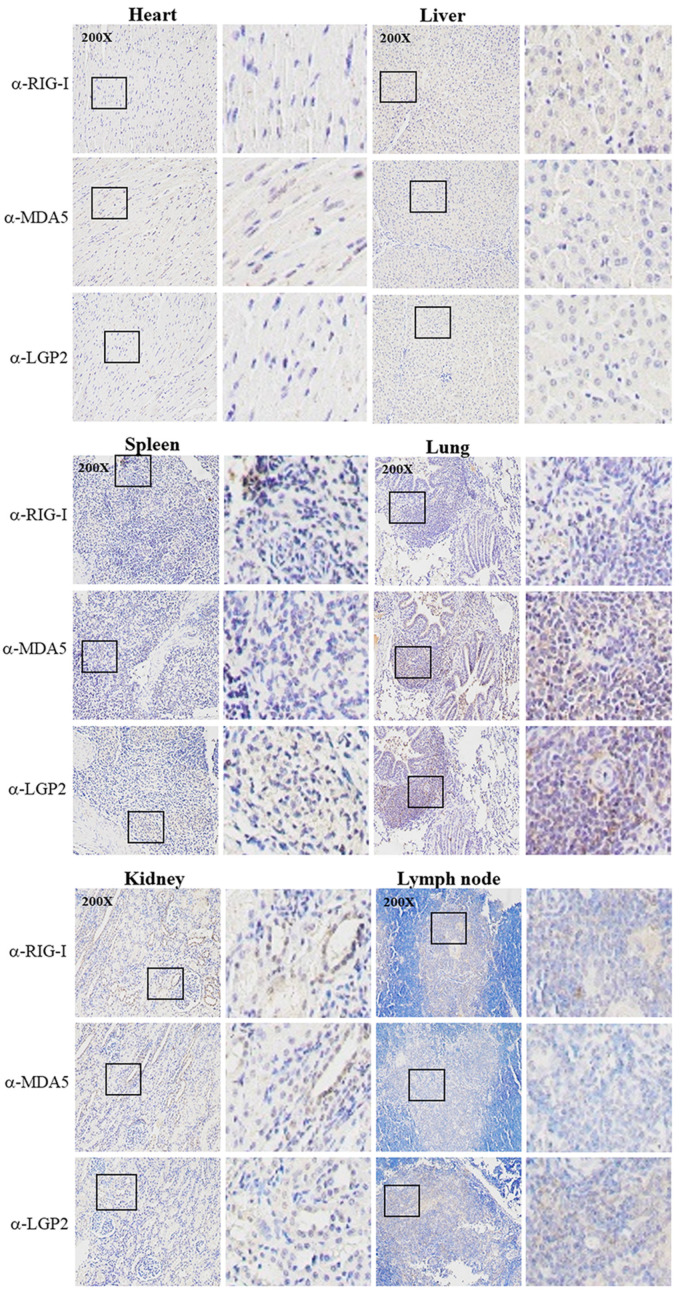
Immunochemistry analysis of RLR expressions in various tissues from PRRSV-infected pigs. Tissues from PRRSV-infected pigs including heart, liver, spleen, lung, kidney and lymph nodes were collected for tissue sectioning. Tissue sections were stained with pRIG-I mAb, pMDA5 mAb and pLGP2 (B9-2) mAb, respectively, followed by hematoxylin counterstaining. The staining results were observed under a microscope at a magnification of 200×, and the boxed areas have been enlarged and are shown on the right of each images. There was no reaction of normal pig tissues with RLR mAbs (not shown).

The three porcine RLR specific mAbs were applied for IHC to detect the RLR distributions and expressions in different tissues from the PRRSV-infected pigs (Figure 7). In the heart, the three RLRs were hardly detected; in the spleen, liver and lymph node, the RLRs were detectable at moderate levels, whereas in the kidney and lung tissues, the RLR expressions were significant, with most of the RLR proteins being in the cytoplasm (Figure 7). In general, the results from IHC based on three RLR mAbs are consistent with those of the RT-qPCR.

## 3. Discussion

RLRs play crucial roles in the protection of hosts, including protecting pigs from virus infections [21]. Here, we developed mAbs that act against porcine RLRs RIG-I, MDA5 and LGP2, which were successfully applied in various immune assays, including Western blotting, immunofluorescence and immunohistochemistry, thus they are useful tools for porcine RLR research. More importantly, one set of RLR mAbs are porcine RLR-specific ones. First, each mAb reacted with its own RLR protein, but it did not cross-react with the two other RLR proteins. This was expected from the amino acid (aa) sequence similarities between the RLR proteins. Even though RLR members share conserved structural organization and functions [22], the aa sequence similarities between the RLR proteins are poor. For porcine RLRs, the aa similarity rates between RIG-I and MDA5, between RIG-I and LGP2 and between MDA5 and LGP2 are 26%, 22% and 28%, respectively. Second, each mAb only reacted with porcine RLR, but not the human RLR counterpart. Porcine RIG-I, MDA5 and LGP2 share 78%, 84% and 82% aa identities with their human RLR counterparts, respectively; therefore, the availability of porcine RLR-specific mAbs demonstrate the fine species specificity that exists between pig RLRs and human RLRs.

There exists one additional small protein during either RIG-I mAb or MDA5 mAb detection (1st–2nd blots, Figure 2). For the porcine RIG-I gene in GenBank, there are six mRNA variants and two protein isoforms, with the smaller RIG-I isoform largely lacking a C-terminal region (~110 aa). Such a small RIG-I isoform should correspond to the small protein bands recognized by anti-RIG-I mAb in Figure 2, which have relative higher amount than full-length RIG-I does. Recently, one report identified a C-terminus truncating variant RIG-I in Japanese eel, and the truncating variant RIG-I was also a signal-active one [23]. For the porcine MDA5 gene in GenBank, there are two protein isoforms, with the smaller MDA5 isoform lacking 47 aa in the CARDs region. Such a small MDA5 isoform likely represents the small protein bands recognized by anti-MDA5 mAb in Figure 2, which have lower amount it than full-length MDA5 does. What are the signaling functions and roles in antiviral immune for these smaller porcine RIG-I and MDA5 isoforms recognized by the corresponding mAbs? Undoubtedly, they deserve to be further investigated.

Even though porcine RLRs and ISG56 proteins are detectable in both PAM and PK15 cells, the protein expressions are higher in PAM than they are in PK15 (Figure 2). This makes sense since PAM is an immune cell, while PK15 is not. However, in PRRSV-infected tissues, the non-immune organ tissues such as those in the lungs and kidneys have higher RLR expression levels than the immune tissues do, including the lymph nodes and spleen. Therefore, this seems to be a dilemma. The reconciliatory explanation may be as follows: Likely, during PRRSV infection, the immune cells in the lung and kidney tissues are enriched and activated to significantly upregulate RLR protein expressions. On the other hand, the tissue resident non-immune cells are also activated and express quite a lot RLR proteins, as we can see in the stimulated PK15 cells. Collectively, the lung and kidney tissues have higher RLR transcript and protein expression levels.

In summary, we acquired a set of porcine RLR specific mAbs, which were successfully subjected to different immune assays, and they are, thus, useful tools in porcine RLR research. Further, by using these RLR mAbs, the unique aspects of porcine RLRs including species specificity, cellular localization and tissue distribution have been revealed, which enriches our understanding of animal innate immunity.

## 4. Materials and Methods

### 4.1. Cells and Reagents

Myeloma Sp2/0, HEK293T and porcine kidney-15 (PK-15) cells were cultured in DMEM (Hyclone Laboratories, Logan, UT, USA) containing 10% fetal bovine serum (FBS) with penicillin/streptomycin. Porcine alveolar macrophages (PAMs, 3D4/21) were cultured in RPMI (Hyclone Laboratories) containing 10% FBS with penicillin/streptomycin. TRIpure Reagent for RNA extraction was from Aidlab (Beijing, China). Restriction endonucleases and T4 DNA ligase (M0203S) were purchased from New England Biolabs (Beijing, China). HiScript^®^ 1st Strand cDNA Synthesis Kit, ChamQ Universal SYBR qPCR Master Mix and 2×Taq Master Mix (Dye plus) were from Vazyme Biotech Co., Ltd. (Nanjing, China). Gateway^®^ LR Clonase^TM^ II Enzyme mix, Donkey anti-Mouse IgG (H+L) Alexa Fluor 488 (R37114), Lipofectamine 2000 and Opti-MEM were from ThermoFisher Scientific (Shanghai, China). Anti-HA mouse mAb (HT301), anti-FLAG mouse mAb (HT201), anti-Actin mouse mAb (HC201), HRP anti-mouse IgG (HS201) and HRP anti-rabbit IgG (HS101) were bought from Transgen Biotech (Beijing, China). Anti-ISG56 rabbit pAb was made in our laboratory. Protino^®^ Ni-TED 2000 Packed Columns was from Macherey-Nagel (Düren, Germany). Montanide gel immunoadjuvant was purchased from SEPPIC SA (Cedex, France). The HT media supplement and Aminopterin were purchased from SIGMA (St. Louis, MO, USA). PEG1500 was purchased from Roche (Shanghai, China). Human IFN-β (D110087-0025) was purchased from BBI life sciences (Shanghai, China). The monoclonal antibody subclass identification kit (C060101) was purchased from CELLWAY-LAB (Luoyang, China). IPTG, PEG20000 and BSA were all purchased from Solarbio life sciences (Beijing, China).

### 4.2. Molecular Cloning, RLR Protein Expressions and Purifications

The prokaryotic and eukaryotic expression vectors for porcine RLRs, RIG-I, MDA5 and LGP2, were prepared as we described previously [12]. The pcDNA pRIG-I CARDs (1-200 aa), pcDNA pMDA5 CARDs (1-200 aa), pcDNA LGP2 L1 (1-200 aa), pcDNA pLGP2 L2 (340-530 aa) and pcDNA pLGP2 L3 (531-685 aa) were all produced in our previous work [12,24]. The pRIG-I-Δ2CARDs (201-943 aa) and pMDA5-Δ2CARDs (201-1023 aa) were amplified by PCR from template plasmids using the primers in Table 1, digested with restriction enzymes and cloned into the *EcoR* I and *EcoR* V sites of the pCAGGS-2HA vector by T4 ligation. The expression plasmids pcDNA human RIG-I-FLAG, pcDNA human MDA5-FLAG and pcDNA human LGP2-FLAG were previously cloned and stored in our laboratory.

The porcine RLR protein expressions in bacteria DE3/BL21 were induced by IPTG. The pRIG-I and pMDA5 proteins in the supernatant of bacterial lysates were purified according to the instructions on the Protino^®^ Ni-TED 2000 Packed Columns Kit, as we described previously [12]. The pLGP2 protein in the precipitation of the bacterial lysates was sonicated and dissolved in an 8 M urea-containing PBS solution at 4 °C overnight and shaken. The overnight suspension was crushed during sonication, centrifuged at 10,000 rpm at 4 °C for 50 min, and the supernatant collected was subjected to urea concentration gradient dialysis and subsequent concentration by PEG 20000. The purified porcine RLR proteins were subjected to SDS-PAGE and stained with Coomassie brilliant blue for qualitative and quantitative identifications.

### 4.3. Immunization, Cell Fusion and Screening of RLR Protein-Specific Hybridomas

Each porcine RLR-purified protein (50–100 μg) was mixed with an immune adjuvant Montanide gel at a volume ratio of 10:1, and the mixture was injected subcutaneously into 6–8-week-old BALB/c mice at multiple points on their back. The second and third immunizations (50 μg and 50 μg proteins mixed with same adjuvant, respectively) were performed at 14 d and 21 d after the first immunization, respectively. Within 2–4 d after the final booster immunization (50 μg protein) via intraperitoneally, the spleen cells from the immunized mice were collected and fused with the Sp2/0 cells at a cell number ratio of 5–10:1 using PEG1500 following the routine procedure. The fused cell mixtures in the HAT medium were dispensed into 96-well cell culture plates containing peritoneal feeder cells, and the plates were placed in a 37 °C, 5% CO_2_ incubator. The growth of the hybridoma cells was observed, and the supernatant was aspirated for antibody detection when the hybridoma clones became significantly large and the supernatant turned yellow. The specificity of antibody secretion in the supernatant of hybridoma cells was detected by ELISA and Western blotting. The specific hybridomas were subcloned via limited dilutions, and the corresponding ascites were prepared according to the standard procedures.

### 4.4. ELISA and Western Blotting

For the ELISA experiment, the hybridoma supernatant was used as the primary antibody; in parallel, the sera of immunized mice and normal mice were used as the positive and negative controls, respectively, whereas PBS was used as the blank control. Each purified porcine RLR protein was the antigen that was used to coat the ELISA plate wells at 4 °C overnight. After three washing steps with PBST, the plate wells were subjected to being patted dry on absorbent paper and blocked in a PBS containing 1% BSA. After washing, the hybridoma supernatants, 1:800 diluted positive and negative sera and PBS were added to the antigen-coated wells at 37 °C for 1.5 h of incubation. Next, HRP goat anti-mouse IgG at a working concentration of 1:5000 was added to all of the wells at 37 °C for 1 h of incubation. Finally, the freshly prepared TMB substrate solution and stop solution were sequentially added, and this was followed by the OD_450_ measurement. S/N values were calculated, and the hybridoma S/N ≥ 2.1 was judged as being positive.

For Western blotting, the 293T cells were transfected with different porcine RLR plasmids with Lipofectamine 2000 for 24 h, or the porcine cells were stimulated with IFNβ for 8–12 h to induce endogenous ISG expressions. The cells were harvested and lysed in RIPA buffer on ice for 10 min. The cell lysates mixed with 1 × SDS sample buffer were boiled at 100 °C for 5–10 min and resolved on 6–10% SDS polyacrylamide gels. The protein bands on the gels were transferred onto PVDF membranes, and the membranes were blocked with 5% non-fat dry milk TBST at pH 7.4. Next, PVDF membranes were sequentially incubated with various primary antibodies and HRP-conjugated goat anti-mouse or anti-rabbit IgG. The protein signals were detected with the enhanced ECL substrate (Tanon, Shanghai, China) and visualized using an Western blot imaging system (Tanon, China).

### 4.5. Immunofluorescence Microscopy

PAM cells were plated in 15 mm glass-bottom cell culture dishes (NEST, China) and cultured at 37 °C in a 5% CO_2_ incubator to 70–80%. The human IFN-β (500 IU/mL, BBI) was directly added to stimulate the cells for 8–12 h. After washing with PBS, the cells were fixed with 5% paraformaldehyde at RT for 10 min, followed by cell permeabilization with 0.5% Triton X-100 for 15 min at RT. The fixed and permeabilized cells were blocked with 5% BSA for 2 h at RT and incubated with the various primary antibodies (pRIG-I mAb, pMDA5 mAb, pLGP2 mAb A1-3 and pLGP2 mAb B9-2). Then, the secondary antibody Donkey anti-Mouse IgG (H + L) Alexa Fluor 488 was added for incubation at RT for 2 h in the dark. After washing them three times with PBST, the cells were counterstained with DAPI (Beyotime, Shanghai, China) at RT for 10 min, and the stained PAM cells were examined under a fluorescence microscope.

### 4.6. Quantitative RT-PCR

Heart, liver, spleen, lung, kidney and lymph node tissues were collected from 10–30-week-old euthanized pigs infected with a high-pathogenic porcine respiratory and reproductive syndrome virus (HP-PRRSV), as well as normal control pigs, and RNAs were extracted with TRIpure reagent from the ground tissues. The extracted RNA was reverse transcribed to cDNA using the HiScript^®^ 1st Strand cDNA Synthesis Kit, and target gene expressions were measured by quantitative PCR (qPCR) using the ChamQ Universal SYBR qPCR Master Mix performed on a StepOne Plus device (Applied Biosystems, Waltham, MA, USA). The qPCR program was denaturation at 95 °C for 30 s, followed by 40 cycles of 95 °C for 10 s and 60 °C for 30 s. The qPCR primers for pRIG-I, pMDA5 and pLGP2 are shown in Table 2. The ΔΔCT method was used to quantify the transcription levels of the porcine RLR genes. 

### 4.7. Immunohistochemistry (IHC)

Tissues from HP-PRRSV-infected pigs including those from the heart, liver, spleen, lung, kidney and lymph nodes were taken for tissue sectioning. After their fixation and dehydration, the tissues were embedded in melted paraffin and subjected to sectioning. The thickness of the tissue sections was 5 μm, and the tissue sections were spread with double-distilled water, loaded on polylysine-coated slides, which were dried at 37 °C overnight, and stored at 4 °C. Before staining, the paraffin sections were sequentially rehydrated, which was followed by antigen retrieval with 3% H_2_O_2_ incubation in the dark to block endogenous peroxidase.

After washing them with PBS, the tissue sections were blocked with 5% BSA at 37 °C for 15 min and incubated with the primary antibodies pRIG-I mAb (1:300), pMDA5 mAb (1:300) and pLGP2 mAb B9-2 (1:500), overnight at 4 °C. The next day, the sections were washed with PBS and incubated with the secondary antibody HRP-conjugated goat anti-mouse (1:200) at RT for 30 min. Then, the specific brown color developed with the substrate DAB for 5 min. After counterstaining with hematoxylin, the tissue sections were dehydrated in gradient alcohols and sealed with neutral resin. The dried tissue sections were subjected to microscopic examination, image acquisition and analysis.

### 4.8. Statistical Analysis

All of the experiments are representative of three similar experiments, and the representative experimental data in graphs are shown as the mean ± SD of triplicate wells. The statistical analysis was performed with the *t*-test where appropriate, which was performed using the software GraphPad Prism 6.0 (San Diego, CA, USA). *p* < 0.05 was considered as statistically significant.

## Figures and Tables

**Table 1:** The PCR primer sequences for cloning of porcine RIG-I-Δ2CARDs and MDA5-Δ2CARDs.

Primer Names	Primer Sequences	Length
RIG-I-Δ2CARDs	F: catcattttggcaaa*GAATTC*atggaggatgacgaaatgaaaacttgt R: tatgggtagctggt*GATATC*ctcaaggttgcccattc	2232 bp
MDA5-Δ2CARDs	F: cattttggcaaa*GAATTC*atgaccgattgctgtgaaagca R: tatgggtagctggt*GATATC*gtcctcatcactagacaaac	2472 bp

Note: The restriction sites are denoted in capitals and italics.

**Table 2:** The qPCR primer sequences for detection of porcine RLR gene transcriptions.

Genes Amplified	Primer Names	Primer Sequences (5′→3′)	Length
pRIG-I	pRIG-I-F	cccagtgtatgagcagcaga	234 bp
	pRIG-I-R	ctggtgttgtggcattcatc	
pMDA5	pMDA5-F	ctcaaagagcatcccctgag	246 bp
	pMDA5-R	gttcgaactctttgcggaag	
pLGP2	pLGP2-F	gttcgaactctttgcggaag	150 bp
	pLGP2-R	gacccttgaactgcttctgc	
pβ-actin	pβ-actin-F	atgaagatcaagatcatcgcg	116 bp
	pβ-actin-R	tcgtactcctgcttgctgatc	

## Data Availability

Not applicable.
